# Tuning Surface Adhesion Using Grayscale Electron-beam Lithography

**DOI:** 10.1021/acs.langmuir.4c00669

**Published:** 2024-07-01

**Authors:** Arushi Pradhan, Luke A. Thimons, Nickolay Lavrik, Ivan I. Kravchenko, Tevis D. B. Jacobs

**Affiliations:** † Department of Mechanical Engineering and Materials Science, University of Pittsburgh, 3700 O’Hara St., Pittsburgh, Pennsylvania 15208, United States; ‡ Oak Ridge National Laboratory, Oak Ridge, Tennessee 37830, United States

## Abstract

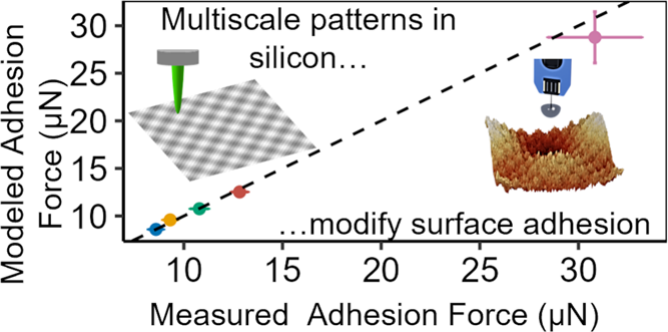

Surface texturing of manufactured products tailors their properties, such as friction, adhesion, biocompatibility, or fluid interactions. However, advancements in this area are largely the result of trial-and-effort testing and generally lack a science-guided framework for determining the surface topography that will optimize performance. The present investigation explores grayscale electron-beam lithography as a means to create multiscale surface patterns to control surface performance. Here, we created and characterized a set of surface textures on a silicon wafer; the textures were superpositions of sine waves of varying wavelengths and amplitudes. First, the multiscale topography of the patterned surface was characterized, using profilometry and atomic force microscopy, to understand its fidelity to the designed-in pattern. The results of this analysis demonstrated how grayscale lithography accurately controlled the lateral size of features but was less precise on the vertical height of the surface, and also introduced inherent roughness below the scale of patterning. Second, a micromechanical tester was used to characterize the adhesion of the surfaces with large-scale polished silicon spheres. The results showed that adhesion could be tailored, with significant contribution from all of the designed-in length scales of topography. The strength of adhesion did not correlate with conventional roughness parameters but could be accurately modeled using simple numerical integration. Taken together, this investigation demonstrates the promise and challenges of grayscale e-beam lithography with multiscale patterns as a method for the tailoring of surface performance.

## Introduction

Surface texturing is widely used in manufacturing to optimize surface performance in both soft- and hard-material applications. The intentional modification of surface topography has been used in technologies from medical devices and robotic grippers to automobile components and equipment for manufacturing automation.^1^ Surface texturing alters key performance metrics, such as adhesion, friction, and wear, by modifying surface properties like the true area of contact between two surfaces, the stiffness of that contacting interface, and how well a surface retains a fluid lubricant.^2^ However, advances in surface texturing are primarily driven by trial-and-error testing and statistical correlations. The field lacks a science-based understanding of how to modify topography to achieve a desired level of performance. Analytical models of surface performance have shown that adhesion and other properties can depend on multiscale topography, in some cases down to nanometer sizes.^3−7^ However, the application of these analytical models to real-world surface texturing has had only limited success, primarily because typical surface texturing contains only a single size scale of topography, both in its creation and in its measurement. This investigation aims to investigate the promise of multiscale surface texturing for adhesion control.

There are two primary approaches to imparting topography to surfaces: adding a rough coating on the surface or selectively removing material from the surface. First, a rough coating can be added, for instance, using chemical vapor deposition (CVD) or physical vapor deposition (PVD). Changes in temperature, pressure, or other deposition parameters cause changes to topography and to the resulting surface properties.^8,9^ A rough coating can also be created by depositing a coating of nanoparticles on the surface. For instance, Nic Spencer’s group demonstrated how nanoparticle coatings control adhesion and friction;^10,11^ others showed their use for biocompatibility^12^ and antireflection/antifogging.^13^ The second method for patterning rough surfaces is to selectively remove material from the surface, either by chemical etching^14^ or by using a laser or lithographic technique to impart patterned features.^15^ For example, adhesion and friction can be reduced by introducing trenches to reduce contact or retain lubricant. Yet both texturing approaches, adding a rough coating or selectively removing a material, lack a framework for rationally tailoring surface performance. Individual investigations show a correlation between surface properties and conventional roughness parameters, such as *R*_a_ and *R*_q_;^14,16,17^ however, these trends typically fail to generalize across conditions. Furthermore, other studies have shown the importance of the shape or spatial distribution of features,^18−20^ which are not captured by conventional roughness parameters. A more robust framework is needed to modify surface patterns to selectively control surface performance.

The present investigation explores grayscale lithography as a means to selectively tailor adhesion. Grayscale lithography creates three-dimensional patterns^21,22^ and is used extensively in micro- and nanoscale optics.^23−26^ While traditional photolithography imparts binary patterns, with only two levels of height, grayscale lithography modulates the exposure dose to create various levels of material removal. Here, grayscale electron-beam lithography (EBL) was used to systematically roughen a silicon surface by superimposing sinusoidal patterns of varying wavelength and amplitude. The topography was then characterized, including below the pixel size of the patterning, to understand fidelity of the surface to the designed pattern. Finally, the effect of patterning on adhesion was analyzed using thousands of contact/pull-off tests with a macroscale silicon sphere. The purpose of this investigation is to advance the understanding of multiscale patterning as a means of tailoring surface performance.

## Experimental Methods

### Creation of Multiscale Patterns in Silicon Surfaces

Grayscale electron-beam lithography^22,27,28^ was carried out on silicon wafers to create a surface morphology with deterministically controlled roughness, as described in Supporting Note S1. Specifically, 200-nm thick poly(methyl methacrylate) (PMMA 495A4, spun at 2000 rpm) was used as the electron-beam resist. The development of the exposed resist was done in a MIBK/IPA (2:1) mixture for a 20 s duration. Once the resist was developed, reactive ion etching (RIE) was used to transfer the grayscale pattern formed in the resist film into the silicon surface, which was done using anisotropic RIE with fluorine chemistry. The designed patterns were 8-bit grayscale (png) images with 2000 × 2000 pixels to govern the e-beam dose at each location in linear dependence to the grayscale level (ranging from 1 to 255). The digital image corresponded to 100 × 100 μm^2^ in physical size on the sample with a pixel size of 50 × 50 nm^2^. The dependence of the residual resist thickness versus electron-beam exposure dose was precalibrated by measuring exposed stairstep patterns, where the exposure dose was varied from the minimum to the maximum levels.

In order to focus on multiscale topography, the patterns comprised the combination of various single-scale topographies—each with a different characteristic size. These single-scale topographies consisted of two-dimensional (2D) sine waves, designated as small (S), medium (M), and large (L), with wavelength and amplitude given in Table 1. The large wavelength was chosen so that at least one complete wavelength was represented in the largest atomic-force-microscopy topography scan, which was 20 μm in size. The small wavelength was set to enable a sufficient resolution (10 pixels, at 50 nm per pixel) to reasonably define a sinewave. The medium wavelength was chosen to be the midpoint, on a log scale, between the large and small wavelengths. The amplitude of the sine waves was designed to have a Hurst exponent of 0.5 (H is the scaling factor related to the fractal dimension) with the total amplitude for all three sinusoids combined to be close to the maximum gray level 255. Additionally, as a reference, we included a “flat-pattern” sample, which was subjected to the same patterning process, but with a designed-in pattern, where every pixel was identical, with a value of 255.

**Table 1:** Wavelength and Amplitude of the Two-Dimensional Sinusoids That Were Superimposed to Create the Various Designed Patterns

2D sinusoids	designation	wavelength (λ)	amplitude (A)
large wavelength	L	19.5 μm	10 nm
medium wavelength	M	3.12 μm	4 nm
small wavelength	S	0.5 μm	1.5 nm

Four arrays of identical patterns were created using the identical recipe, in a single processing batch. The adhesion measurements were done on a different array than the topography measurements to ensure that the contact-based topography measurements, such as stylus profilometry and atomic force microscopy, would not deform the surface. White-light interferometry images of all arrays were taken to ensure consistency across all arrays in the batch.

### Characterization of the Multiscale Patterns

After creation, the true topography of each pattern was characterized across length scales. For the smallest scales, atomic force microscopy (AFM) was used in tapping mode (Bruker Dimension Icon VI). Measurements were collected with scan sizes from 20 μm down to 200 nm. To prevent wear, DLC-coated probes (Tap DLC 300, BudgetSensors BudgetSensors, Sofia, Bulgaria) were used, with an average tip radius of 15 nm, as characterized in a transmission electron microscope. Larger-scale topography was measured using a stylus profilometer (Alpha Step IQ, KLA Tencor, Milpitas, CA), with line lengths ranging from 450 μm down to 50 μm. The radius of the stylus probe was 5.29 μm, as characterized in a scanning electron microscope. Finally, a scanning white-light interferometer (Contour GT-I, Bruker, Billerica, MA) was used to take two-dimensional images with edge lengths ranging from 600 μm down to 50 μm (corresponding to lens configurations with nominal magnifications from 5× to 100×). For each tool, various measurements were performed spanning the largest and smallest sizes that could be reasonably measured. Three replicates were performed at each scan size, each in a new location on the sample.

### Adhesion Measurements

**Figure 1:**
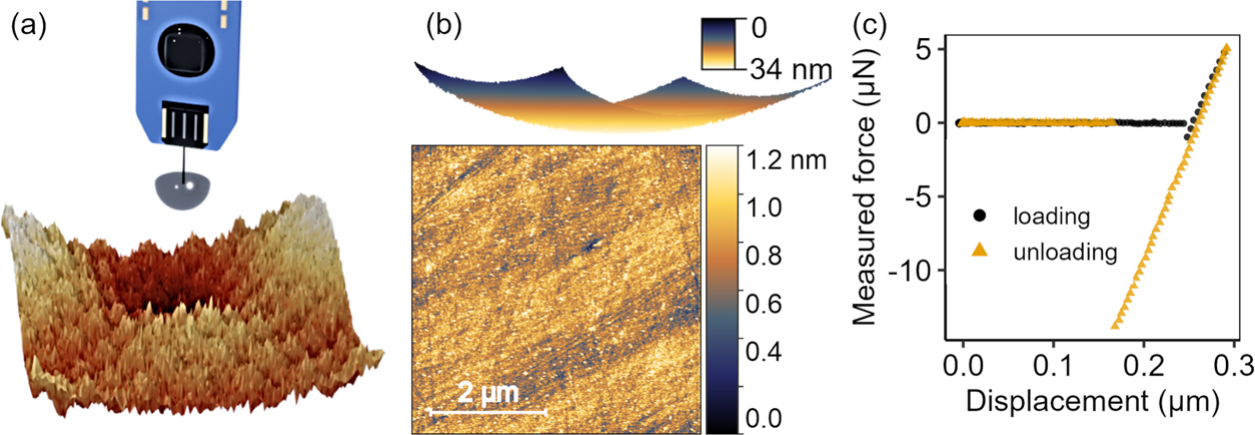
Large-scale adhesion tests were performed on lithographically created surfaces to analyze the effect of patterning on silicon–silicon adhesion. (a) Contact and pull-off tests were performed using a stick–slip piezo positioner (not shown) and a MEMS-based force sensor. 0.5-mm silicon hemispheres were used to mimic the large-scale adhesion that would be measured in, e.g., a semiconductor-manufacturing context. (b) The hemisphere surfaces were prepolished and imaged using AFM (top); when the curvature is digitally removed (bottom), the remaining roughness has a root-mean-square deviation of less than 1 nm. (c) More than 400 tests were performed on each sample; during each test, the real-time force was measured during loading (black) and unloading (gold).

Adhesion measurements were performed using 0.5-mm silicon hemispheres (SI00-SP-000105, Goodfellow Corp., Coraopolis, PA) mounted on a MEMS-based force-sensing probe (FT-MA02, FemtoTools, Buchs, Switzerland) as shown in Figure 1. Prior to mounting, the spheres were polished from their as-received state using a 0.05-μm alumina polishing suspension (as described in Supporting Note S2). The spheres were polished as smooth as possible, achieving an RMS height of <1 nm, as measured using AFM with a scan size of 5 μm as shown in Figure 1b. Adhesion measurements were carried out using a velocity during the approach and withdrawal of approximately 30 nm/s. For adhesion tests on patterns, the sphere was loaded upon contact up to 5 μN (corresponding to a nominal Hertzian pressure of 31 MPa). For adhesion tests on the flat-pattern sample, the sphere was loaded upon contact up to 50 μN (corresponding to a nominal Hertzian pressure of 67 MPa) due to a typo in test setup. However, other testing on these large 0.5-mm spheres demonstrated only a 5% difference in adhesion with this level of variation in preload. The present tests were performed in a 20-by-20 array for each pattern, and a fresh location of each pattern was contacted for each contact test. Prior to testing, samples were cleaned using an IPA solution in a sonicator. The process for mounting the silicon sphere onto the probe is described in Figure S1 of the Supporting Information.

The adhesion experiments were carried out in an environmental chamber, where dry nitrogen continually flowed through the test chamber to reduce humidity and capillary formation, and the relative humidity was measured in the chamber as <2% RH, which was the minimum measurement of the humidity gauge. A static elimination bar was also used (ionizing bar EI PS, Haug Static, Mississauga, ON) to minimize the buildup of electrostatic charge, e.g., from contact electrification or from the flowing gas.

Despite efforts to keep the sphere and sample clean throughout the process, there are some opportunities for contamination. The sphere could potentially pick up contamination during the mounting process when it contacts a cleaned glass slide or during positioning of cameras inside the testing chamber when dust particles might enter the chamber. In the case where the probe did occasionally pick up some contamination during testing, it resulted in all-zero adhesion measurements after contamination occurred. In such tests, the presence of contamination was confirmed using optical interferometry, and all adhesion values after the abrupt drop to zero were removed from consideration.

## Results and Discussion

### Analysis of the Patterned Topography and Assessment of Grayscale e-Beam Lithography for Multiscale Surface Texturing

**Figure 2:**
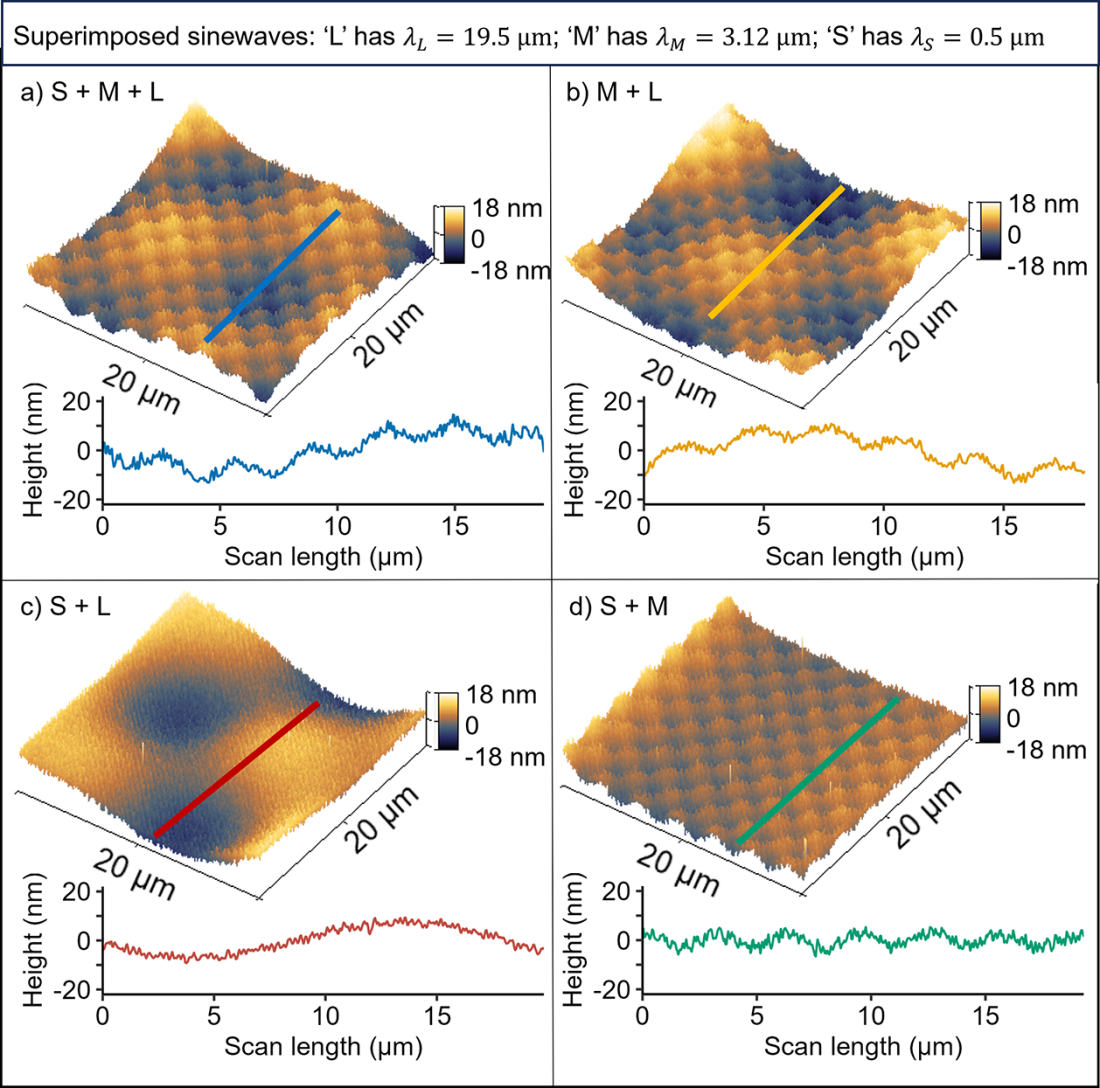
Surfaces were textured using multiscale patterns comprising the superpositions of sine waves of different sizes. The true topography of the fabricated surfaces was characterized across 6 orders of magnitude using atomic force microscopy (this figure) as well as stylus and optical profilometry (Figure S2 of the Supporting Information). The area scans (top of each panel) and line scans (bottom) show the different combinations of small (S), medium (M), and large (L) sine waves, whose wavelength and amplitude are defined in Table 1.

Four multiscale surfaces were created and characterized as shown in Figure 2 (and as described in Section S3 of the Supporting Information). Each surface is designated by its combination of individual sine waves, for instance, “S + M + L” contains a superposition of all three sine waves, while “S + L” contains only the small and large wavelengths. The AFM scans are shown in Figure 2, while the stylus profilometry and optical interferometry data are shown and discussed in Supporting Note S3. The patterned surface shows that the two-dimensional sinusoids create checkerboard-like patterns at different scales, which have been superimposed on one another.

Measurements taken from AFM clearly reveal all scales of sinusoids on the height profile of the surfaces. All of the scales of roughness are present in the S + M + L surface. In the other surfaces, it is easiest to notice by eye which sinusoid is missing from each one. The large-wavelength sinusoid (L) is visible on all surfaces except S + M (Figure 2d). It has a period (or wavelength) corresponding to approximately the scan size of these images (20 μm), matching the designed-in period. The maximum peak-to-valley variation of the designed S + M + L surface should be 31 nm (twice the sum of the amplitudes of the individual sine waves); however, the measurements of the corresponding surface showed a peak-to-valley variation of 20.4 nm, which is significantly smaller. In fact, the measured peak-to-valley height is lower than designed for all four patterns. This reflects the fact that the etch depth at each pixel does not scale exactly linearly with exposure dose. While a precalibration was performed to compensate for this difference, all sinusoids still showed a variation from the designed-in amplitude.

The medium-scale sinusoid (M) is visible on all surfaces except for S + L (Figure 2c). It has a period of approximately one-sixth of the scan size shown in Figure 2, or approximately 3 μm, once again corresponding to the designed-in period. The surface S + M has a peak-to-valley height of approximately 10.1 nm, which is slightly different from the designed-in value of 11 nm, for reasons discussed above. Finally, the small-scale roughness appears simply as noise in Figure 2. It is more clearly visible in smaller-size AFM scans (see Figure S3 of the Supporting Information). It is not suppressed as much as expected in the M + L scan, as will be discussed in a subsequent section. Overall, the comparison of designed vs actual topography yields the first important insight for the use of grayscale lithography to optimize surface performance: while the lateral scales of features are precisely controlled, the vertical scale is often less accurate.

**Figure 3:**
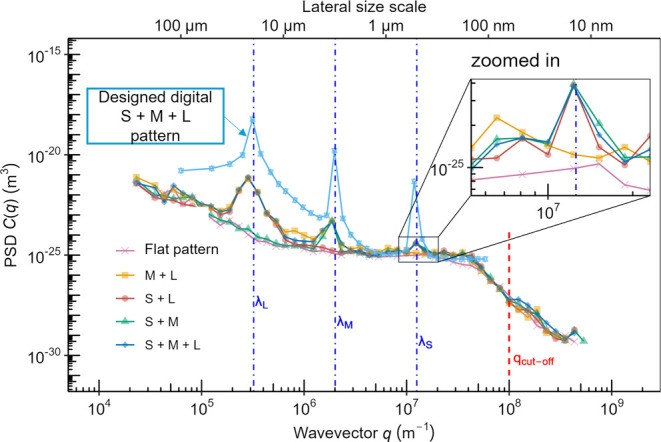
Scale-dependent topography analysis shows the contribution to topography from each size scale, enabling comparisons among the created surfaces and against the designed-in surface. The true topography of the fabricated surfaces was characterized across 6 orders of magnitude using AFM and profilometry (main text). At least 48 measurements were taken from each patterned surface; all measurements were combined, with no adjustable parameters, using the power spectral density. PSDs of each individual measurement are shown in Supporting Note S5; this figure shows only the averaged PSD for each surface, facilitating comparisons between surfaces.

In order to combine all data from a single sample, taken at various scan sizes and with a variety of instruments, the power spectral density (PSD) was used.^29^ The PSD is a mathematical tool that separates out the contributions to topography from different size scales. The PSD is computed as the square of the Fourier transform of the height profile in the distance domain. The PSD (*C*(*q*)) of all measurements is presented as a function of wavevector , where λ represents lateral size scale (wavelength) in real space. The PSD is shown in Figure 3, with the frequency-space wavevector on the bottom *x*-axis and the real-space lateral size scale (wavelength) on the top *x*-axis. A reliability analysis is performed for each surface characterization technique^30^ to assess the impact of resolution limits and tip-radius artifacts; then, the unreliable data was removed. The PSD was computed for each individual measurement, and these were combined into a single surface descriptor by taking the average at each wavevector. The PSD analysis was performed using the open-source contact.engineering software (described in ref (30) and available at https://contact.engineering).

Scalar roughness parameters can also be computed to describe the topography in a single number. Specifically, the root-mean-square (RMS) height *h*_rms_, the RMS slope *h*′_rms_, and the RMS curvature *h*″_rms_ were calculated as the zeroth, second, and fourth moment of the PSD, respectively^29,31^
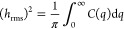
1
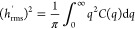
2
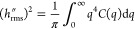
3When these parameters are computed in frequency space, the integration bounds determine the included scales; therefore, unlike real-space calculations which are limited to a single measurement, these parameters can include contributions from all scales of measured topography. The all-scale values of these roughness parameters are shown in Table 2. As expected, all patterns containing the large ‘L’ sinusoid have larger RMS height than the ones that do not, because *h*_rms_ is most sensitive to large-scale features. Likewise, all patterns containing the small ‘S’ sinusoid have larger RMS curvature than the ones that do not, becaus*e* is most sensitive to small-scale features.

**Table 2:** RMS Parameters of All Surfaces

types of patterns	*h*_rms_ (nm)	*h*′ _rms_ ()	*h*″_rms_ (μm^–1^)
M + L	5.12 ± 0.10	0.036 ± 0.001	1.52 ± 0.05
S + L	4.90 ± 0.11	0.041 ± 0.001	1.75 ± 0.05
S + M	2.42 ± 0.07	0.039 ± 0.001	1.70 ± 0.06
S + M + L	5.32 ± 0.12	0.038 ± 0.001	1.61 ± 0.06
flat pattern (gray level 255)	2.03 ± 0.13	0.028 ± 0.001	1.30 ± 0.09

Clearly visible in the power spectral density are the three designed-in peaks. In Figure 3, these are labeled as λ_L_, λ_M_, and λ_S_. This allows the precise determination of their wavevector and corresponding wavelength; as discussed above, these accurately match the designed-in period. Furthermore, according to Parseval’s law, there must be correspondence between the features observed in real space and that of frequency space; so it makes sense that the visible “checkerboards” in Figure 2 correspond to visible peaks in Figure 3.

**Figure 4:**
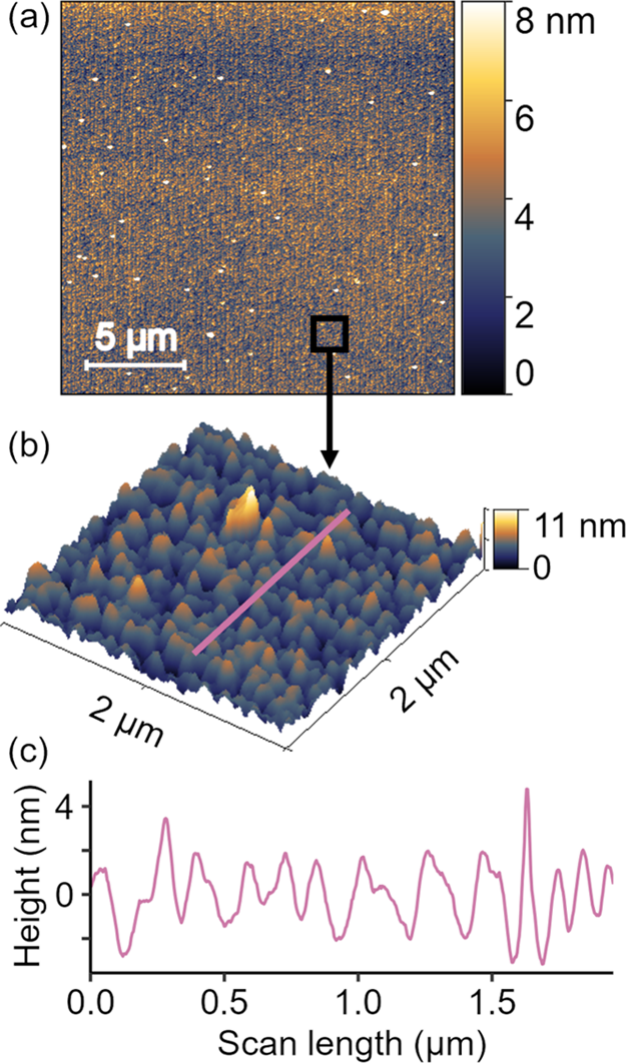
The flat-pattern sample, which is patterned to be nominally flat, reveals the inherent roughness of the patterning technique. This surface was patterned in the same batch as the others, but the designed-in pattern contained a single height (gray level (exposure level) = 255) at every pixel. These atomic force microscopy measurements demonstrate the roughness that is inherent to the grayscale e-beam lithography process. (The relationship between panels is schematically showing their relationship as zoom and cross-section; they do not represent the exact same locations on the sample.) The observed topography likely reflects the inherent roughness of the etching technique or corresponds to a characteristic size of the polymer resist.

The PSD also showed a substantial background roughness that was imparted on the surface unintentionally. The construction of the digital surface had no additional topography outside of the three sine waves. Furthermore, this background roughness is also present on the flat-pattern sample (topography shown in Figure 4 and in Supporting Note S4), which had a perfectly flat designed-in topography, where all pixels shared the same gray level of 255. In all cases, including the sinusoidal surfaces and the flat-pattern sample, there is a clear bilinear background signal with a kink at *q* = 3.14 × 10^–7^, which corresponds to 200 nm in lateral scale. Subpixel roughness has been observed in other contexts,^28,32^ yet the physical origin is still unclear. Here, the small-scale roughness is not believed to arise from contamination during patterning because industry-standard practices were used to process and clean the wafers before and after patterning (Supporting Note S1). Instead, it likely arises due to the inherent morphology of the PMMA polymer, either in its natural state or roughness that develops during the development or etching stage. It is noteworthy that the relevant lateral size of 200 nm corresponds approximately to the radius of gyration for PMMA with a molecular weight of 495 kDa.^33^ However, the radius of gyration of a polymer (*R*_g_) in solvent can be only indirectly related to the small-scale spatial inhomogeneity of polymer in a solid film. The formation of a solid PMMA film by spin coating of its solution is a seemingly simple process, but at the molecular level, it involves a complex transition of PMMA macromolecules from dissolved in solution to densely packed on the silicon surface during solvent evaporation. Therefore, *R*_g_ can only be a semiqualitative predictor of the scale, where intrinsic small-scale inhomogeneities in a polymer film are present. This inherent roughness is clearly distinguishable from the small-wavelength S peak that was designed-in. Yet, the inherent roughness and the S sinusoid have a similar contribution to overall adhesion reduction.

There is also an inherent roughness to the EBL patterning at larger scales (wavelength greater than 100 μm). These large-scale features may arise due to heterogeneities in exposure profile across patterns, or so-called “proximity effects,” where the height of one region can be affected by the height of neighboring regions due to the physics of development and etching, or due to inherent long-wavelength roughness of the silicon wafer after etching, e.g., due to inhomogeneities in the drying and removal of the liquid etch. This large-scale roughness is readily apparent in the WLI images shown in Figures S2 and S4 of the Supporting Information. Upon further optimization of the lithography process, the very largest scales of topography could be slightly reduced (Figure S5 of the Supporting Information), but the overall large-scale background could not be eliminated.

Overall, the evaluation of measured topography using a power spectral density yields the second important insight for the use of grayscale lithography to optimize surface performance: there can be significant roughness from the lithography itself, which will be superimposed on any designed-in pattern, and this inherent roughness will have different characters at the scales of the polymer molecule and at larger scales.

### Analysis of the Resulting Adhesion and Assessment of Grayscale e-Beam Lithography for Tuning Surface Adhesion

**Figure 5:**
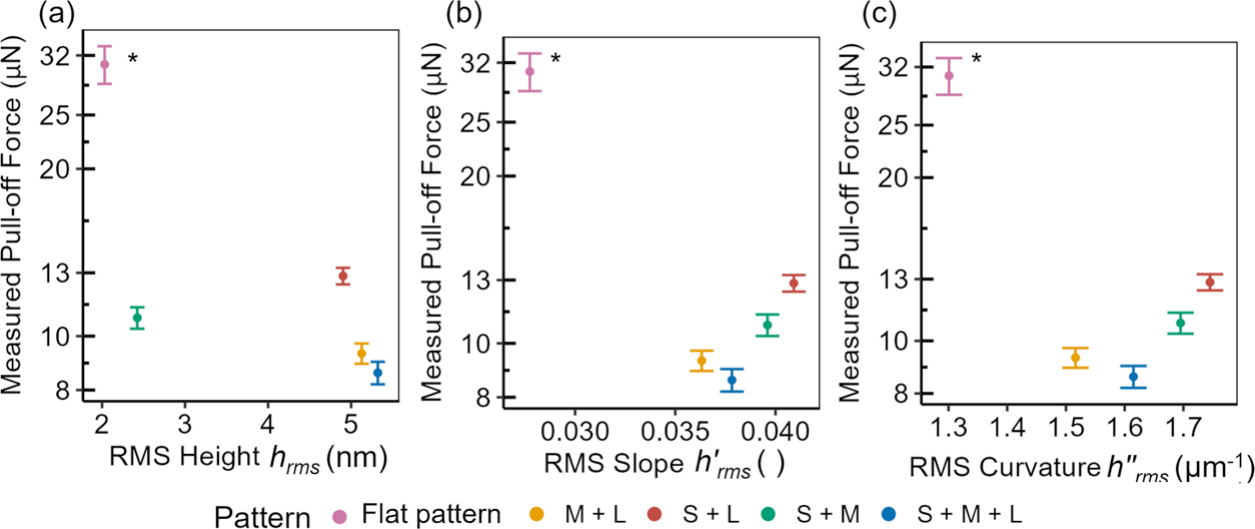
Adhesion force does not correlate with simple scalar metrics for roughness. Pull-off force is plotted against scalar roughness parameters, as calculated in Table 2. No simple trends are observed between adhesion and (a) RMS height, (b) RMS slope, or (c) RMS curvature. The coloring of data points is by pattern and is consistent with prior figures. The asterisk (*) denotes that the flat-pattern adhesion experiment was performed with a higher preload than the others, but this is not expected to explain the significant difference in adhesion shown here (see Experimental Methods section).

Figure 5 shows the results of more than 400 measurements of adhesion on each surface. As described in the Experimental Methods section, the contact/pull-off tests were performed with millimeter-scale silicon spheres, with each pull-off test performed in a new location. The magnitude of adhesion differs between the different patterns. The lowest adhesion values were produced by the combination of all sine waves (called S + M + L), which had an average adhesion of 8.59 ± 0.40 μN. The M + L pattern produced an adhesion of 9.31 ± 0.39 μN. The S + M pattern produced an adhesion of 10.79 ± 0.48 μN. Finally, the S + L pattern produced an adhesion of 12.83 ± 0.44 μN. As described, the flat-pattern sample was included as a baseline of a patterned surface with no designed-in topography. It had the least roughness but contained the inherent roughness of the patterning technique. The adhesion on the flat pattern (30.83 ± 2.4 μN) was the highest, as expected. ANOVA analysis, with 95% confidence, showed a statistically significant difference between all surfaces except S + M + L and M + L (see Supporting Note S6).

No simple trends exist for adhesion as a function of root-mean-square topography parameters. The pull-off force does not scale with any scalar metric, in conflict with models and experiments, suggesting simple relationships with RMS height^6,34^ or RMS slope.^35^ Classic multiasperity models, such as that of Greenwood and Williamson,^36^ paired with subsequent analyses by Nayak^37^ and later McCool,^38^ suggest a dependence on RMS curvature. However, none of these models can be fit to the measured data.

In the absence of simple trends, we analyze results using a “brute-force” numerical integration approach. This is widely used in the contact-mechanics literature^39^ and by some of the present authors.^40^ In short, a pairwise interaction potential was computed pixel-by-pixel across the two contacting surfaces. The interaction potential determines the adhesive energy (and thus adhesive force) acting between corresponding pixels as a function of their separation distance. By integrating over all pixels in the top and bottom surface, the result is the total force acting between them. By repeating this procedure at various values of separation distance between the sphere and plane, the full force–separation curve can be computed. The lowest (i.e., most negative) value found at any distance represents the pull-off force.

The contacting surfaces were represented using AFM scans of both the mm-scale hemisphere and each of the various patterned surfaces. We used the largest (20 μm) AFM scans, with a corresponding pixel size of approximately 40 nm. To account for subpixel roughness, we superimposed random small-scale roughness statistically identical to each pattern; specifically, computer-generated random topography was created, using a Fourier-filtering algorithm,^41^ based on the small scales (8–80 nm) of the measured PSD (Figure 3) for each pattern. The topography of the hemisphere was created by stitching together 5-μm AFM scans of representative silicon spheres (as shown in Figure 1b) and superimposing these on a 250-μm-radius spherical shape. Multiple different hemisphere scans were used in each calculation to account for the variation in the level and type of polish imparted on different spheres.

An exponential interaction potential was used to describe the adhesive energy *U*, as is common in contact calculations^42^
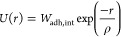
4where *W*_adh,int_ is the intrinsic work of adhesion between the materials, *r* is the vertical spacing of a pair of pixels, and ρ is the range of interaction. For a small value of ρ, the potential is very deep and short range; a larger value of ρ stretches the potential to be weaker at any specific distance but to act over longer distances. Plasticity was captured using a simple rigid-perfectly-plastic approximation at each pixel, with a chosen hardness of 13 GPa.^43^ In each numerical simulation, the hemisphere was loaded up to the level of preload used in its corresponding experiment.

**Figure 6:**
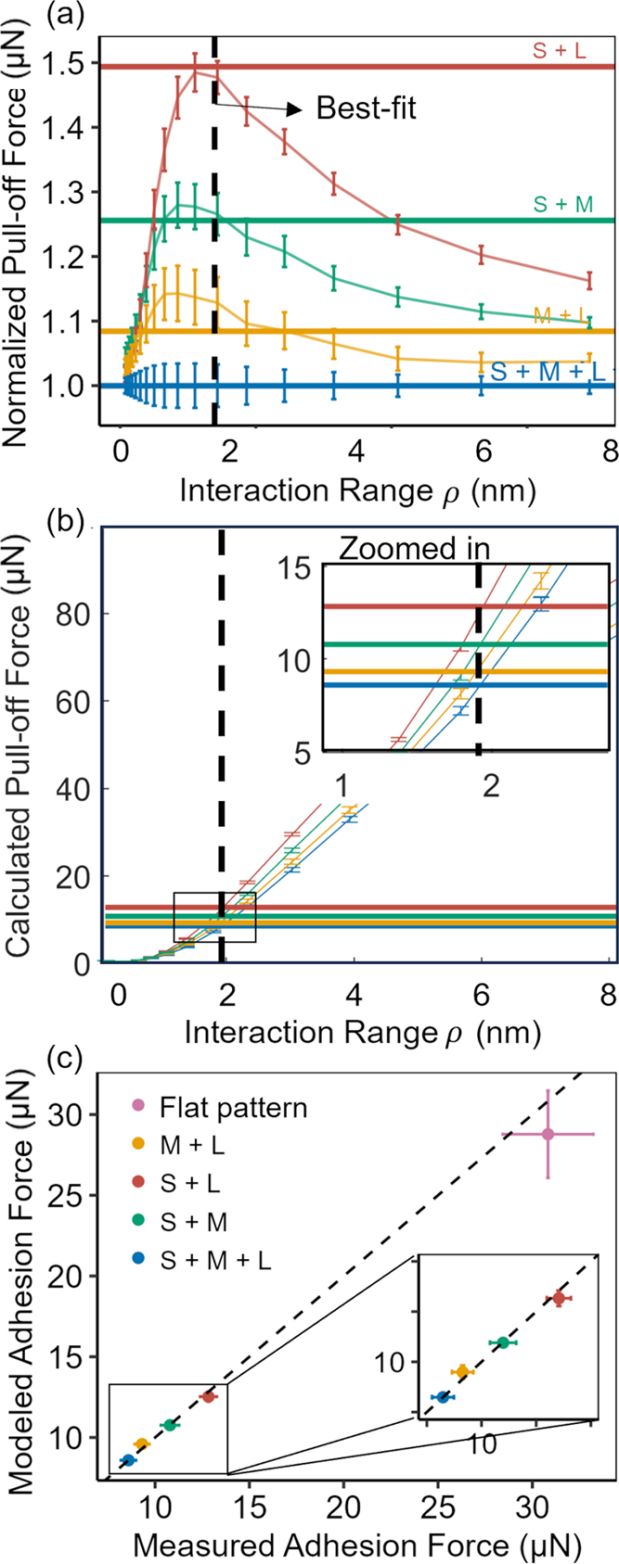
Numerical integration combines the patterned topography and the adhesive interactions to accurately describe the measured adhesion. Numerical integration is used to compute a pull-off force from topography and interaction parameters. A numerical fitting routine was applied to adhesion measurements to extract best-fit parameters for (a) the range of interaction (also called the “length-scale of adhesion”) and (b) the work of adhesion (also called the “adhesive energy”). Once the interaction parameters are known, the same numerical integration can be used to predict the adhesion on any topography. (c) The modeled adhesion force is compared against the measured value, with the dashed black line representing perfect equivalence. When this model is extrapolated to the flat-pattern sample (pink, not used in the fitting), the model agrees with the measured adhesion within 7% error.

The fit between the numerical modeling and the measured adhesion for all surfaces is shown in Figure 6. The thick, horizontal lines represent the mean adhesion for each of the sinusoidal patterns. The thin lines with error bars represent the calculated value of adhesion for different values of the range of interaction ρ (*x*-axis). By first fitting to the normalized pull-off force, we eliminate *W*_adh,int_ such that ρ is the only fit parameter. Then the absolute adhesion values are used (Figure 6b), enabling the direct fitting for work of adhesion. Taken together, the work of adhesion was calculated as 118.3 ± 5.9 mJ/m^2^ and the range of interaction as 1.92 ± 0.60 nm.

The measured work of adhesion is reasonable for van der Waals adhesion between passivated silicon surfaces. While silicon interfaces can form covalent bonds resulting in very large adhesion, as used in direct wafer bonding, the adhesion has been shown in ref (44) to be reduced by an order of magnitude with surface passivation by hydrogen or hydroxyl groups. It is expected that the present surfaces will be fully terminated due to significant air exposure. Therefore, the measured value of 118 mJ/m^2^ aligns with prior understanding. The measured range of adhesion is larger than typical estimates for van der Waals interactions of 0.3–0.6 nm.^45^ These longer-than-expected ranges of adhesion have been observed in prior investigations (e.g., see refs (40,46−49)), but the precise origin is still being investigated. An extensive discussion of longer-than-expected ranges of adhesion was included in ref (40). In the present investigation, similar effects are envisioned: plasticity, contact electrification, large contribution from near-to-contact regions (described by Casimir interactions), or capillary interactions from surface-adsorbed water. While the adhesion experiments were performed in a dry nitrogen (<2% RH) environment, we cannot rule out capillary effects from trace water. We avoided the use of hydrophobic surface treatments to avoid any unintended modifications to the surface topography. Instead, by performing all adhesion measurements under a consistent environment, we hoped to isolate the specific effects of surface topography. Further work would be required to test the relative contributions from different effects.

To test the accuracy of the numerical fitting procedure, it was extrapolated to the flat-pattern surface to evaluate the accuracy of the prediction. The numerical fitting was applied only for the sinusoidal surfaces (the training data set) without considering the flat-pattern sample (the test data set). The same model, including best-fit interaction parameters, is used to compute the expected adhesion on the flat-pattern sample. Remarkably, the computed adhesion agreed with the experimental data within 7% error.

More generally, in a manufacturing context, it is useful to compute the “effective work of adhesion” *W*_adh,eff_, to describe how the patterned topography changes the large-scale value that is relevant for applications. The effective work of adhesion for a given material system is known to vary widely with topography.^50,51^ Using a simple contact-mechanics analysis^47^ based on the nominal size of the hemisphere (*R* = 250 μm), the effective work of adhesion can be computed from the adhesive force *F*_adh_ as
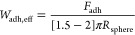
5where *R*_sphere_ is the nominal radius of the large-scale sphere (against a nominally flat substrate, with ). The range of prefactors in the denominator reflects the different contact-mechanics models that are commonly applied.^51^ This approach yields computed values of effective work of adhesion ranging from *W*_adh,eff_ = [20 – 26] mJ/m^2^ for the flat-pattern surface, down to *W*_adh,eff_ = [6 – 7] mJ/m^2^ for the S + M + L surface, varying by more than a factor of 3 with the designed-in pattern. In a manufacturing context, this *W*_adh,eff_ would be used to predict the large-scale adhesion performance, whereas the numerical simulation enables the determination of *W*_adh,int_ for any given set of materials and conditions. Thus, patterns can be designed to modulate the strength of the surface adhesion.

Despite the advancements, there are several limitations to the present study. First of all, the research plan was limited to a single material (silicon spheres on patterned silicon surfaces), created using a single lithographic technique. It is therefore not clear how well the results will extend to other materials and other approaches. Second, for maximum clarity on the effects of different size scales, the designed-in patterns were limited to well-defined sinusoidal shapes with discrete combinations of wavelengths and amplitudes. Further work would be required to determine how results from these artificial surfaces generalize to real-world materials, which typically contain a continuous array of overlapping topographies, often spanning an even larger range of size scales. Finally, while prior work has demonstrated the measurement of topography to the atomic scale,^52^ these techniques were not practical to apply to the present samples due to the difficulty of cross-sectioning these very small (600 μm in lateral size) patterns; therefore, the very smallest-scale topography is unknown.

## Conclusions

By designing, fabricating, measuring, and characterizing a set of patterned surfaces, we have explored the tailoring of surface adhesion by using multiscale patterns. Grayscale electron-beam lithography was used to pattern multiscale sinusoids into silicon surfaces, and then their corresponding adhesion was measured against a silicon countersurface. A comprehensive topography characterization revealed precise control over the lateral scales of features but only moderate control over the vertical scale, due in part to inherent roughness that is introduced by the patterning technique itself. The mechanical testing revealed a successful variation of surface adhesion by more than a factor of 3, with effective adhesion energies varying from approximately 6–20 mJ/m^2^. The origin of the deviation was linked to the patterned topography using a simple numerical integration technique, which can also be used to design future patterns to achieve a specific value of surface adhesion.

## Data Availability

All data associated with this manuscript is publicly available. Data from adhesion measurements and calculations have been deposited in the University of Pittsburgh’s online repository, D–Scholarship. It is accessible via DOI at http://dx.doi.org/10.18117/81b7-x102. All topography measurements have been made publicly available through Contact.Engineering^30^ and are accessible via DOI by using the following links: Patterned Surface S + M + L (https://doi.org/10.57703/ce-jvcqq); Patterned Surface S + M (https://doi.org/10.57703/ce-rtq5j); Patterned Surface M + L (https://doi.org/10.57703/ce-whc88); Patterned Surface S + L (https://doi.org/10.57703/ce-5kzzp); Flat-Pattern Sample (https://doi.org/10.57703/ce-ybtnr); and Silicon Spheres (https://doi.org/10.57703/ce-6jquf).

## Supporting Information Available

Additional information about the process of grayscale patterning; additional details on creation of hemispheres for adhesion testing, including a visual description of the process for creation and processing for adhesion testing; additional topographic characterization of the multiscale patterns, including using white-light interferometry, stylus profilometry, and additional data from atomic force microscopy; complete PSD characterization for each multiscale pattern; additional representations of the adhesion data and ANOVA analysis (PDF)
