# Supplementary material for: Tuning Surface Adhesion Using Grayscale Electron-beam Lithography
Source: Langmuir. 2024 Jul 1;40(28):14257–65. doi: 10.1021/acs.langmuir.4c00669 (PMC11256748; doi:10.1021/acs.langmuir.4c00669)
Supplement: Supplementary file 1 — la4c00669_si_001.pdf [file la4c00669_si_001.pdf]

Supporting Information for the Paper Titled:

Tuning Surface Adhesion Using Grayscale Electron-beam Lithography

Arushi Pradhan, Luke A. Thimons, Nickolay Lavrik, Ivan I. Kravchenko, Tevis D.B. Jacobs.

Supporting Note S1. Additional Information About the Process of Grayscale Patterning.

The primary steps of the grayscale patterning process are as follows:

- **Deposition of the PMMA layer on a silicon wafer.**
  - Good adhesion of this layer to silicon is due to physisorption of PMMA macromolecules onto the silicon surface.
- **E-beam exposure and subsequent development.**
  - During development, the solvent dissolves and removes partially-scissored PMMA chains from the substrate. The fraction of the scissored PMMA molecules increases with the exposure dose. As a result, the remaining PMMA thickness decreases with the dose.
- **Transfer of the profile formed in the PMMA into silicon by reactive ion etching.**
  - During this step the PMMA, of variable thickness, acts as a sacrificial masking material that is mostly consumed by the end of the etching process. Any remaining residue of PMMA is removed by oxygen plasma.

The final oxygen-plasma cleaning step is commonly used in wafer-scale processing to remove any residual polymer or organic contamination from the surface of silicon substrates after lithographic patterning. Because we rely on well-established protocols to minimize residual contamination of the silicon surface, it is reasonable to assume that the sample surfaces are represented by silanol surface chemistry typical for silicon with a native oxide, both before and after patterning. Subsequent contamination is possible, and even likely, to occur during sample transport and storage; however, the samples are assumed to be extremely clean during lithographic patterning.

Supporting Note S2. Additional Details on Creation of Hemispheres for Adhesion Testing

The detailed process of preparing hemispheres for the adhesion testing is shown in Supplemental Figure S1. First, silicon spheres with a diameter of 0.5 mm are affixed to a polishing holder and ground flat on one side using polishing paper (steps 1a-b, in Supplemental Figure S1). Subsequently, the hemispheres are detached from the holder using acetone, and any debris is removed using IPA solution(steps 1c-d). The hemisphere is then mounted onto a flat screw using crystal bond and inspected with a white light interferometer (WLI) to verify their shape and ensure a radius of 0.25 μm (step S1e). Following this, the hemispheres undergo polishing with a 0.05-μm Al suspension for 2 minutes, followed by cleaning in an IPA solution using a sonicator (steps 2a-b). If significant contamination is present after polishing, a Kim wipe dipped in IPA solution is used to mechanically remove debris (step 2c). The samples then undergo a second WLI inspection to ensure they are well-polished and free of contaminants, after which they are removed from the screw with acetone and cleaned again by sonication in IPA solution (steps 2d-e). Next, the hemispheres are positioned apex-down on a clean glass slide with a small amount of crystal bond

on the side (step 3a). The slide is heated to 80°C on a hotplate. The micromechanical tester with force sensing probe is positioned above the slide. The probe is dipped into a small amount of crystal bond and then lowered onto the hemisphere. The probe with mounted hemisphere sample is again imaged in the WLI to confirm its cleanliness and proper mounting before being returned to the tester (step 3b). The tester is then placed inside a vacuum chamber with the patterned grayscale samples (step 3c). Ultra-high purity nitrogen gas is flowed to reduce relative humidity to less than 2%, and after a half-hour equilibration period, the adhesion test is conducted in a 20 x 20 grid pattern. Upon completion of the test, the hemisphere is detached from the probe by dipping in acetone (step 3d). The hemisphere is then mounted onto a flat puck using double-sided table and imaged using AFM (step 3e).

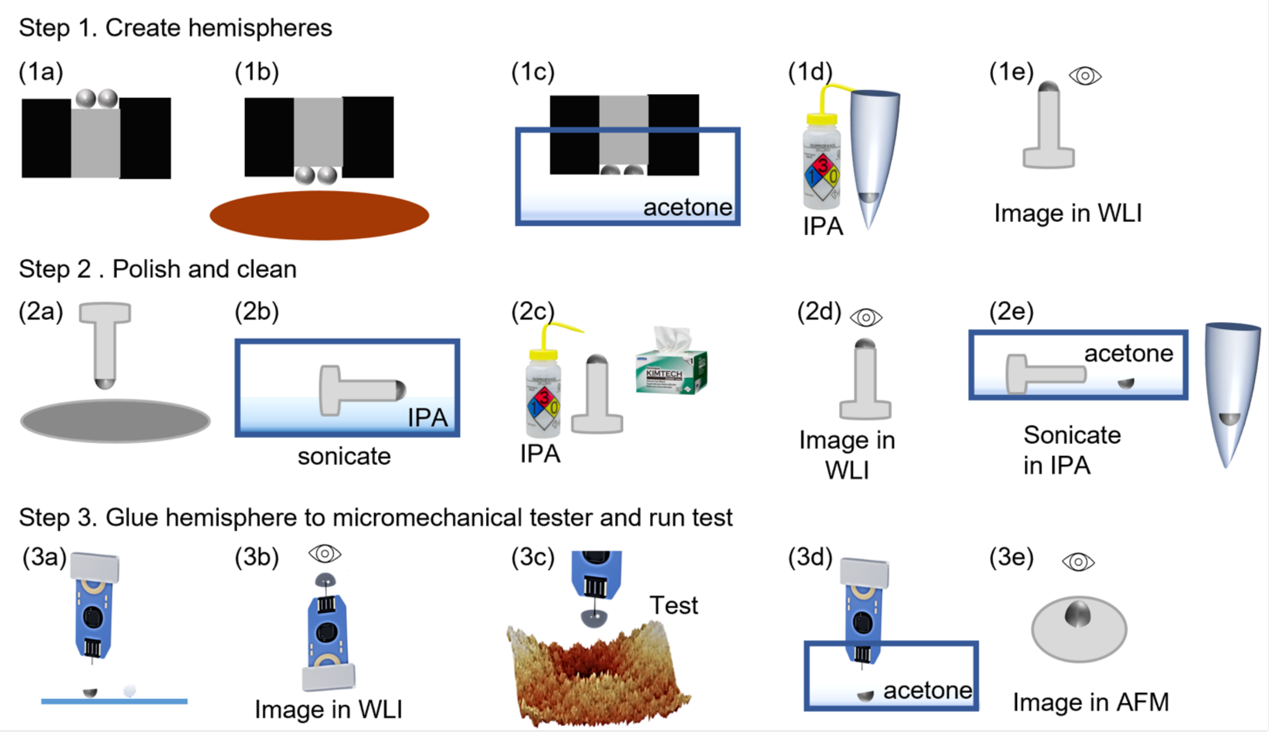

Supplemental Figure S1. Detailed process of sample creation and processing for adhesion test. See text for description of each step.

<u>Supporting Note S3.</u> Additional Topographic Characterization of the Multiscale Patterns

The large-scale topography was characterized using white-light interferometry (WLI) and stylus profilometry (Supplemental Figure S2). Multiple measurements were taken at magnifications ranging from 5X to 100X with the WLI, with representative images shown in Figure S2a-d. Stylus measurements were taken with scan lengths ranging from 500 µm to 50 µm; the sinusoidal features are visible in the line scans shown in Figure S2e. The WLI images show large-scale roughness features across all samples, discussed further in the main text and in Supporting Note S, below.

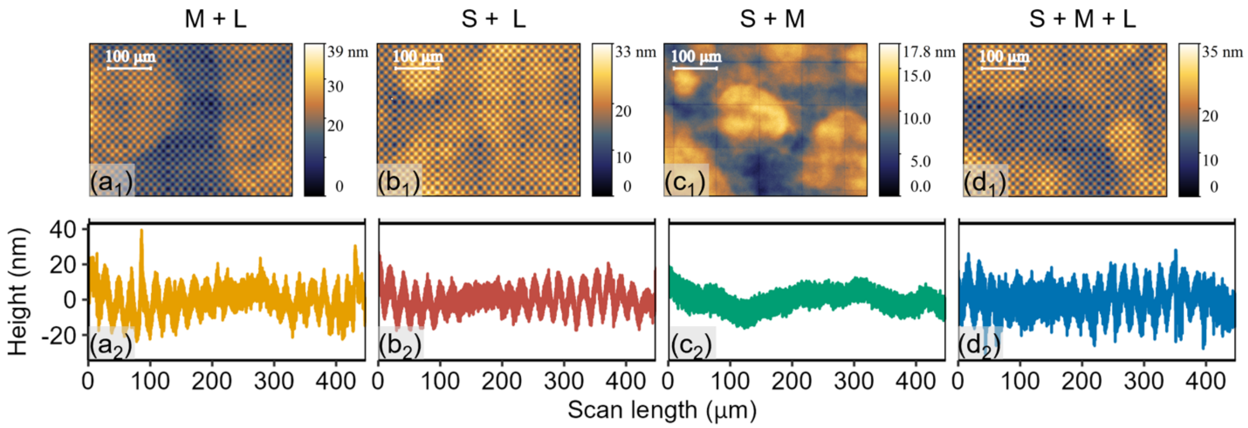

Supplemental Figure S2. Large scale roughness of patterned surfaces characterized using white light interferometer (WLI) and stylus profilometer with 5.29 µm probe.

In addition to the AFM topography measurements shown in the main text, even smaller-size AFM scans (Supplemental Figure S3) reveal the roughness that was present at the very smallest scales. As described in the main text, the smallest-scale sinusoid (S) is difficult to distinguish by eye from the inherent roughness; however they can be readily distinguished using multiscale analysis (see the power spectral density in Figure 3).

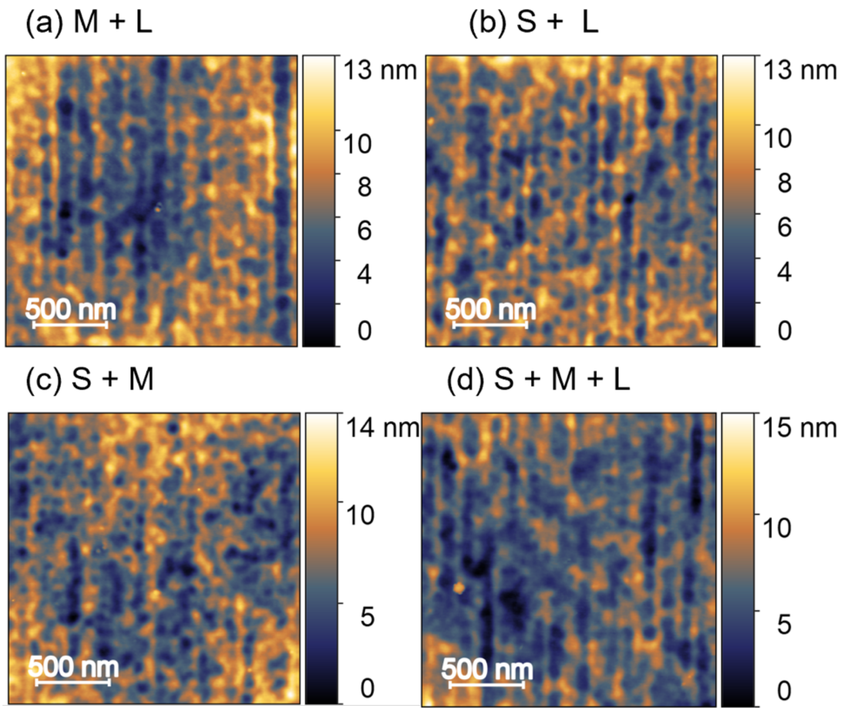

Supplemental Figure S3. AFM scans at 2 µm for the patterned surfaces show similar small-scale roughness. Representative 2 µm scans for the four patterns show similar roughness at the small scales.

<u>Supporting Note S4.</u> Topographic Characterization of Other Relevant Surfaces.

The WLI and stylus measurements for the flat-pattern sample (Supplemental Figure S4) also show roughness variation at the largest scale with roughness features that are almost a hundred microns in size. This is similar to what was observed in the sinusoidal surfaces. As discussed in the main text, the origins of this large-scale roughness could be due to factors such as the inherent roughness of the silicon wafer, due to inhomogeneity in drying and removal of the liquid etchant, proximity effects during patterning, or due to unequal exposure profiles across pixels.

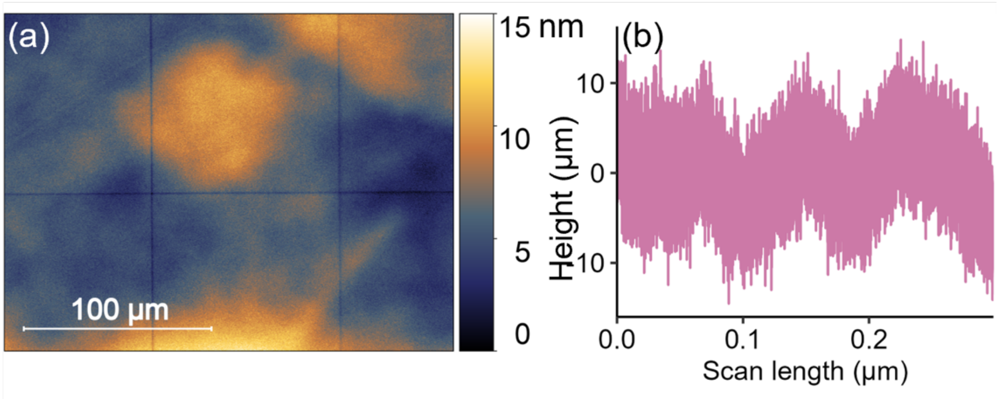

Supplemental Figure S4. Representative images of (a) WLI and (b) stylus profilometer measurements for the flat-pattern sample.

In subsequent patterning, we showed that these features can be reduced through optimization of the lithography process (Supplemental Figure S5). However, this large-scale roughness could not be eliminated entirely.

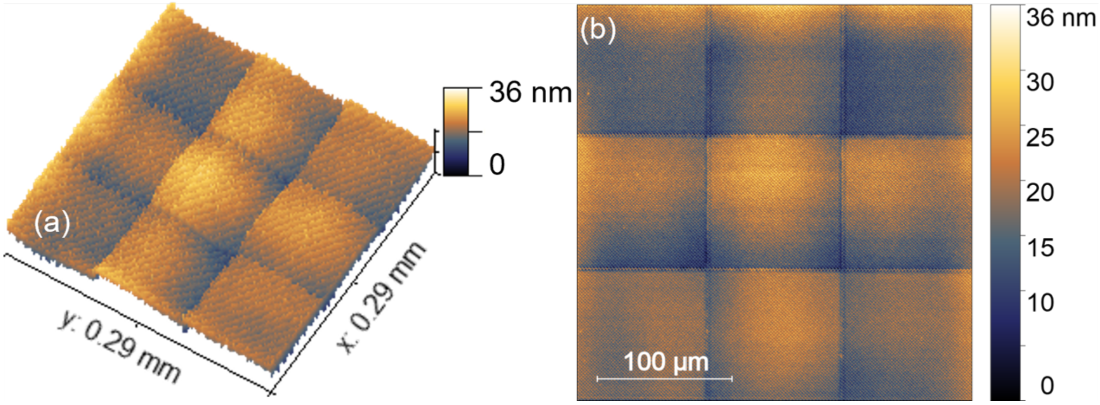

Supplemental Figure S5. WLI measurements of a later version of the patterned sample, created by grayscale lithography with the identical designed-in pattern, but small variations in process.

Supporting Note S5. **Complete PSD Characterization for Each Multiscale Pattern**

While Figure 3 of the main text shows only the averaged PSDs that describe each surfaces, Supplemental Figure S6 shows the individual PSDs that are calculated directly from each topography measurement. The individual PSDs are shown as points, with colors representing the technique, while the black lines represent the averaged data (which is what appears in Figure 3). It should be noted that all of the individual PSDs agree with one another, even across techniques, without the use of any adjustable parameters. In all cases, the various techniques and various magnifications agree within the measurement uncertainty.

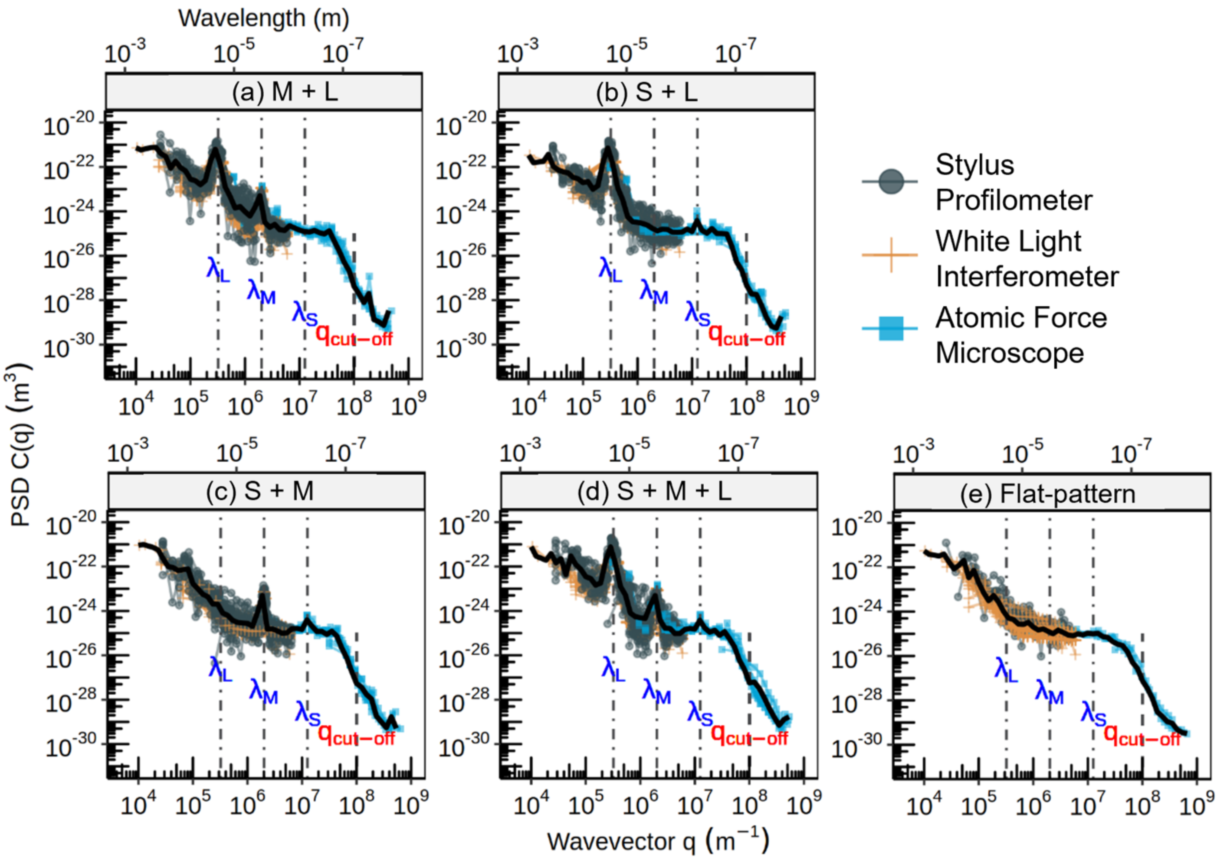

Supplemental Figure S6. Individual PSD curves from different measurements are averaged to create a comprehensive description of the surface across six orders of magnitude. The different instruments are indicated by color; grey for stylus profilometer, gold for white light interferometer and blue for AFM measurements. The measurements are cut-off at the resolution of the instrument used. For probe-based techniques the cut-off is computed (main text) from the size of the probe used (5.29 µN for stylus profilometer and 20 nm for AFM). The WLI measurements are cut-off at the lateral resolution of the technique (approximated as 1 µm). The solid black line is the average across all three techniques. The dashed blue lines indicate the size scales of the designed-in sine waves, with large (λ_L), medium (λ_M) and short (λ_S) wavelengths. (See Table 1 of the main text for specific wavelengths and amplitudes of each designed-in sine wave.) The dashed red line indicates the cut-off applied to the PSD after which the AFM scans are affected by instrument noise.

<u>Supporting Note S6.</u> Additional Representations of the Adhesion Data and ANOVA Analysis

The adhesion data is represented as histograms in Supplemental Figure S7, and the results of an ANOVA analysis are shown in Supplemental Table S1.

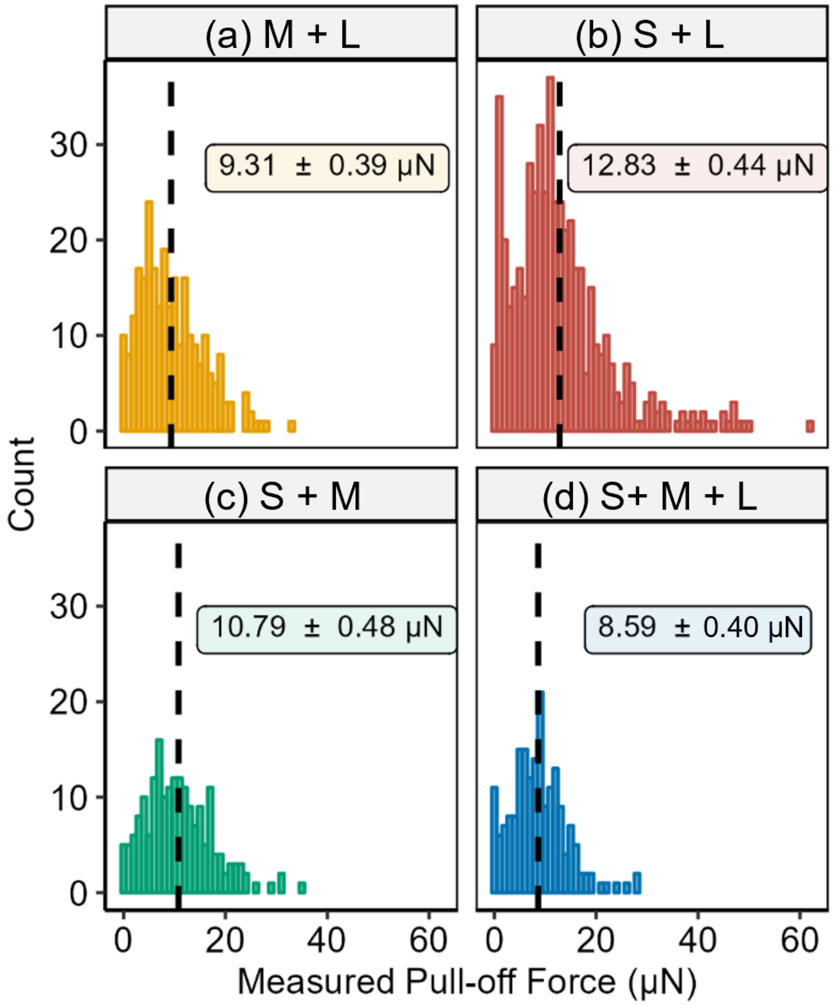

Supplemental Figure S7. Histograms of the measured pull-off force for the four patterns are shown. The dotted line represents the average measured pull-off force. The highest adhesion is seen in pattern S + L while the lowest adhesion is seen in sample with all wavelengths S+M+L.

Supplemental Table S1: ANOVA analysis on adhesion measurements

Pattern 1	Pattern 2	p-values	Significance
M + L	S + L	2.60×10^{-9}	significant
M + L	S + M	0.017	significant
M + L	S + M + L	0.19	not significant
S + L	S + M	0.0016	significant
S + L	S + M + L	1.70×10^{-12}	significant
S + M	S + M + L	0.00043	significant

An ANOVA analysis with 95% confidence level was performed on the pull-off forces measured for the patterned samples. The results show that the difference in adhesion is significant for all samples except M+L and S+M+L. These two surfaces are statistically indistinguishable; all other pairs of surfaces show meaningful differences in adhesion.
